# Differential prognostic impact of cytopenic phenotype in prefibrotic vs overt primary myelofibrosis

**DOI:** 10.1038/s41408-022-00713-6

**Published:** 2022-08-12

**Authors:** Giacomo Coltro, Francesco Mannelli, Giuseppe Gaetano Loscocco, Carmela Mannarelli, Giada Rotunno, Chiara Maccari, Fabiana Pancani, Alessandro Atanasio, Alessandro Maria Vannucchi, Paola Guglielmelli

**Affiliations:** 1 Department of Clinical and Experimental Medicine, University of Florence, Florence, Italy; 2 Center of Research and Innovation for Myeloproliferative Neoplasms (CRIMM), Azienda Ospedaliero-Universitaria Careggi, Florence, Italy

**Keywords:** Myeloproliferative disease, Translational research

**Dear Editor**,

Cytopenias are frequent and distinctive features of primary myelofibrosis (PMF). Anemia is the most common, has consistently been associated with shortened survival, and is an integral component of prognostic models (IPSS, DIPSS/-plus MIPSS70/-plus) [1–4]. Albeit less frequent, also thrombocytopenia (defined as a platelet count <100 × 10^9^/L) was included in the DIPSS-plus and MIPSS70/-plus scores as independent predictor of reduced survival [3–7]. Conversely, leukopenia is the least frequent and has been inconsistently associated with inferior survival [8–10].

Overall, the balance between myeloproliferative and myelodysplastic traits in PMF results in two main clinical phenotypes that are characterized by distinct peripheral blood (PB) presentations: patients with features of myeloproliferation exhibit elevated cell counts, mainly leukocytes and platelets (proliferative phenotype), while patients exhibiting myelodysplastic traits present with cytopenias involving one or more hematopoietic lineages (cytopenic phenotype [CP]) [11, 12]. Although not strictly defined, the CP has been associated with poor prognosis, but cytopenias have been usually considered individually [12].

In the current study, we aimed at investigating the phenotypic and prognostic correlates of a CP in a large cohort of PMF patients, with a specific focus on the distinction between prefibrotic (pre-) and overt PMF. Cytopenias were defined as follows: leukopenia for leukocytes <4 × 10^9^/L, sex-adjusted anemia for hemoglobin (Hb) <11 g/dL for male and <10 g/dL for female, and thrombocytopenia for platelets <100 × 10^9^/L. A CP was defined by the presence of at least one cytopenia, whereas patients not included in the cytopenic group were considered as having a proliferative phenotype. Sex-adjusted anemia was further categorized as moderate (Hb 9–10.9/8–9.9 g/dL for male/female, respectively) and severe (Hb < 9/8 g/dL for male/female, respectively). Similarly, moderate and severe thrombocytopenia was defined by platelets 50–99 × 10^9^/L and <50 × 10^9^/L, respectively. Patients with severe anemia and/or thrombocytopenia were considered as having a severe CP. Details on methods are reported in Supplemental Information.

A total of 431 patients with WHO-defined PMF were included in the study, 216 (50%) pre-PMF and 215 (50%) overt PMF. Patients’ characteristics according to PMF diagnosis are listed in Supplemental Table 1. In pre-PMF, leukopenia, sex-adjusted anemia and thrombocytopenia were found in 12 (6%), 40 (19%), and 18 (8%) patients, respectively. The corresponding figures in overt PMF were 29 (13%), 92 (43%), and 30 (14%), respectively (Fig. 1A). Overall, a CP was identified in 50 (23%) and 105 (49%) patients with pre- and overt PMF, respectively (*P* < 0.0001). Patients with a severe CP were 22 (10%) in pre-PMF and 42 (20%) in overt PMF (*P* < 0.0001), while the corresponding figures for the presence of ≥ 2 cytopenias were 15 (7%) and 39 (18%), respectively (*P* < 0.0001). Table 1 reports the comparison of proliferative *versus* cytopenic phenotypes in pre- and overt PMF, separately.

**Fig. 1:**
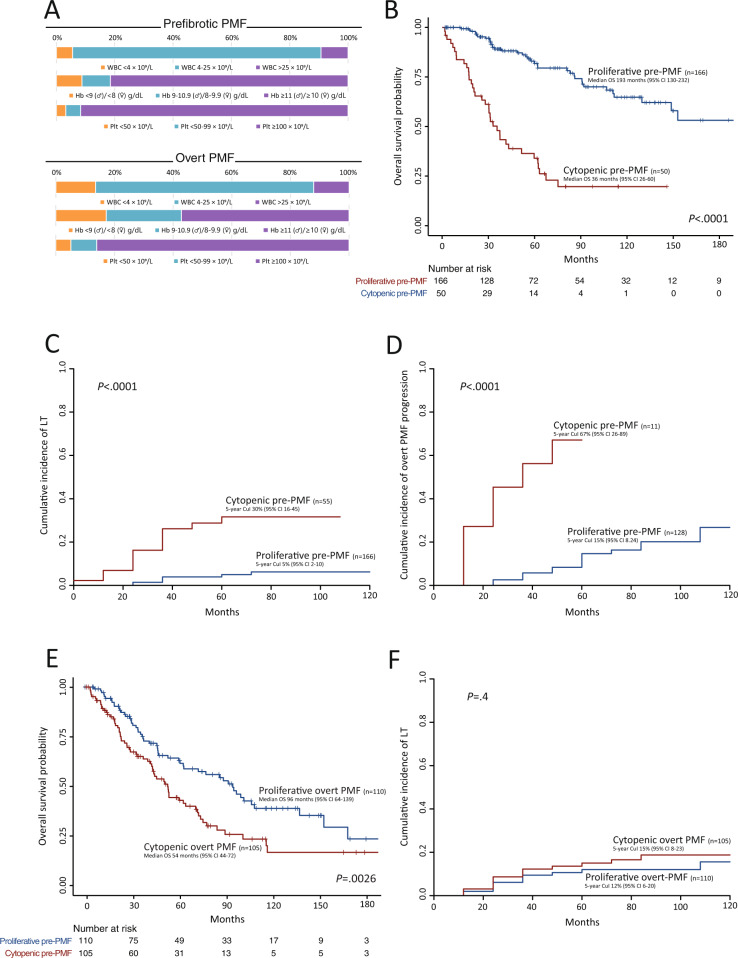
Characteristics and outcomes of patients with prefibrotic and overt PMF according to disease phenotype (cytopenic vs proliferative). **A** Bar graph reporting the distribution of peripheral blood cell counts in pre-PMF (top) and overt PMF (bottom). **B** Kaplan-Meier estimates of overall survival in patients with pre-PMF according to disease phenotype (cytopenic vs proliferative). **C** Competing risks-adjusted estimates of cumulative incidence of leukemic transformation in pre-PMF according to disease phenotype (cytopenic vs proliferative). **D** Competing risks-adjusted estimates of cumulative incidence of progression to overtly fibrotic phase in 139 pre-PMF patients according to disease phenotype (cytopenic vs proliferative). **E**. Kaplan-Meier estimates of overall survival in patients with overt PMF according to disease phenotype (cytopenic vs proliferative). **F** Competing risks-adjusted estimates of cumulative incidence of leukemic transformation in overt PMF according to disease phenotype (cytopenic vs proliferative). Abbreviations: CI confidence interval, CuI cumulative incidence, Hb hemoglobin, LT leukemic transformation, OS overall survival, Plt platelets, pre-PMF prefibrotic primary myelofibrosis, WBC white blood cells.

**Table 1:** Clinical and laboratory features of patients with WHO-defined prefibrotic and overt PMF stratified by the disease phenotype (cytopenic versus proliferative).

Variable		Prefibrotic PMF			Overt PMF		
		Proliferative pre-PMF *n* = 166 (77%)	Cytopenic pre-PMF *n* = 50 (23%)	Proliferative vs cytopenic pre-PMF *P* value	Proliferative overt PMF *n* = 110 (51%)	Cytopenic overt PMF *n* = 105 (49%)	Proliferative vs cytopenic overt PMF *P* value
Clinical and demographics	Male sex; *n* (%)	81 (49)	37 (74)	**0.0017**	76 (69)	80 (76)	0.24
	Age at diagnosis, years; median (range)	56 (18–90)	68 (24–89)	**<0.0001**	59 (21–83)	67 (34-89)	**<0.0001**
	Peripheral CD34 + , %; mean (SD); evaluable = 138/140	0.2 (1.1)	0.7 (1.2)	**0.0015**	1 (1.6)	1.8 (3.7)	0**.0155**
	PB blasts, %; mean (SD); evaluable = 215/208	0.2 (0.9)	1.5 (3)	**<0.0001**	0.7 (1.6)	1.4 (3)	0.18
	LDH, U/L; median (range); evaluable = 158/156	308 (127–2521)	464 (146–2643)	**0.0030**	614 (194–1919)	690 (130–2981)	0.26
	BM fibrosis grade 1 (pre-PMF)/3 (overt PMF); *n* (%); evaluable = 210/199	116 (73)	45 (90)	**0.0107**	29 (29)	42 (43)	0**.0373**
	Splenomegaly (>5 cm below the LCM); *n* (%); evaluable = 212/207	67 (41)	30 (61)	**0.0132**	86 (80)	76 (76)	0.45
	Hepatomegaly; *n* (%); evaluable = 205/202	27 (17)	22 (47)	**<0.0001**	36 (34)	42 (43)	0.19
	Constitutional symptoms; *n* (%); evaluable = 196/202	27 (17)	16 (43)	**0.0005**	33 (32)	44 (44)	0.07
MPN drivers	*JAK2* mutated; *n* (%); evaluable = 197/202	118 (74)	19 (50)	**0.0036**	72 (69)	57 (58)	0.10
	* JAK2*^V617F^ AB; median (range); evaluable = 131/126	35 (1–100)	43 (1–68)	0.11	44 (9–95)	38 (5–100)	**0.0347**
	* JAK2*^V617F^ AB lower quartile; *n* (%); evaluable = 131/126	36 (32)	4 (21)	0.33	8 (11)	16 (29)	**0.0149**
	*CALR* mutated; *n* (%); evaluable = 196/198	29 (18)	4 (11)	0.28	24 (23)	16 (17)	0.26
	*MPL* mutated; *n* (%); evaluable = 196/200	8 (5)	3 (8)	0.50	3 (3)	8 (8)	0.11
	Triple negative; *n* (%); evaluable = 196/197	9 (6)	1 (32)	**<0.0001**	5 (5)	15 (16)	**0.0115**
	Double mutated; *n* (%); evaluable = 195/196	5 (3)	1 (3)	0.88	2 (2)	1 (1)	0.61
Myeloid neoplasm-associated genes	*ASXL1* mutated; *n* (%); evaluable = 176/182	17 (12)	10 (28)	**0.0203**	36 (38)	38 (44)	0.36
	*CBL* mutated; *n* (%); evaluable = 156/162	3 (2)	2 (7)	0.23	6 (7)	7 (9)	0.57
	*CSF3R* mutated; *n* (%); evaluable=111/105	1 (1)	0 (0)	0.71	1 (2)	0 (0)	0.38
	*CUX1* mutated; *n* (%); evaluable = 105/96	0 (0)	1 (9)	**0.0033**	0 (0)	2 (5)	0.11
	*DNMT3A* mutated; *n* (%); evaluable = 156/164	5 (4)	3 (10)	0.18	9 (10)	3 (4)	0.11
	*EZH2* mutated; *n* (%); evaluable = 176/182	3 (2)	1 (3)	0.82	16 (17)	12 (14)	0.61
	*IDH1/2* mutated; *n* (%); evaluable = 176/182	0 (0)	1 (3)	0.05	6 (6)	8 (9)	0.44
	*KIT* mutated; *n* (%); evaluable = 138/140	3 (3)	0 (0)	0.44	0 (0)	1 (2)	0.27
	*NF-E2* mutated; *n* (%); evaluable = 132/131	4 (4)	1 (4)	0.88	3 (4)	3 (5)	0.77
	*N/KRAS* mutated; *n* (%); evaluable = 137/139	2 (2)	3 (13)	**0.0084**	7 (9)	13 (21)	0.06
	*RUNX1* mutated; *n* (%); evaluable = 138/139	0 (0)	2 (9)	**0.0014**	3 (4)	3 (5)	0.84
	*SETBP1* mutated; *n* (%); evaluable = 111/105	0 (0)	3 (23)	**<0.0001**	1 (2)	1 (2)	0.86
	*SF3B1* mutated; *n* (%); evaluable = 137/141	5 (4)	1 (4)	0.99	6 (8)	6 (9)	0.74
	*SH2B3/LNK* mutated; *n* (%); evaluable = 136/141	2 (2)	2 (9)	0.07	6 (8)	1 (2)	0.08
	*SRSF2* mutated; *n* (%); evaluable = 176/182	10 (7)	6 (17)	0.08	9 (9)	13 (15)	0.24
	*TET2* mutated; *n* (%); evaluable = 157/163	27 (21)	7 (23)	0.80	14 (16)	15 (19)	0.59
	*TP53* mutated; *n* (%); evaluable = 139/143	2 (2)	2 (8)	0.08	2 (3)	3 (5)	0.49
	*U2AF1* mutated; *n* (%); evaluable = 137/141	0 (0)	1 (4)	**0.0255**	3 (4)	10 (16)	**0.0165**
	*ZRSR2* mutated; *n* (%); evaluable = 111/105	8 (8)	2 (15)	0.39	2 (3)	5 (11)	0.13
	HMR mutations^║^; *n* (%); evaluable = 176/182	24 (17)	11 (31)	0.08	44 (46)	49 (57)	0.13
	≥2 HMR mutations^†^; *n* (%); evaluable = 176/182	6 (4)	6 (17)	**0.0086**	21 (22)	18 (21)	0.88
Cytogenetics	Abnormal karyotype; *n* (%); evaluable = 163/136	23 (18)	15 (44)	**0.0013**	30 (38)	19 (33)	0.49
	Favorable karyotype; *n* (%)	120 (93)	22 (65)	**<0.0001**	61 (78)	44 (76	0.72
	Unfavorable karyotype; *n* (%)	8 (6)	4 (12)		13 (17)	9 (16)	
	Very high-risk karyotype; *n* (%)	1 (1)	8 (24)		4 (5)	5 (9)	
Prognostic stratification	IPSS risk stratification; evaluable = 193/195						
	Low risk; *n* (%)	84 (54)	4 (11)	**<0.0001**	34 (34)	9 (9)	**<0.0001**
	Intermediate-1 risk; *n* (%)	54 (35)	7 (19)		37 (37)	15 (16)	
	Intermediate-2 risk; *n* (%)	10 (6)	9 (24)		18 (18)	31 (32)	
	High risk; *n* (%)	8 (5)	17 (46)		10 (10)	41 (43)	
	DIPSS risk stratification; evaluable = 193/195						
	Low risk; *n* (%)	84 (54)	4 (11)	**<0.0001**	34 (34)	9 (9)	**<0.0001**
	Intermediate-1 risk; *n* (%)	64 (41)	10 (27)		55 (56)	21 (22)	
	Intermediate-2 risk; *n* (%)	8 (5)	19 (51)		10 (10)	51 (53)	
	High risk; *n* (%)	0 (0)	4 (11)		0 (0)	15 (16)	
	MIPSS70 risk stratification; evaluable = 172/171						
	Low risk; *n* (%)	96 (71)	3 (8)	**<0.0001**	8 (8)	2 (3)	**0.0002**
	Intermediate risk; *n* (%)	33 (24)	20 (56)		59 (65)	33 (41)	
	High risk; *n* (%)	7 (5)	13 (36)		24 (26)	45 (56)	
	Deaths; *n* (%)	40 (24)	36 (72)	**<0.0001**	54 (49)	64 (61)	0.08
	Leukemic transformation; *n* (%)	7 (4)	13 (30)	**<0.0001**	13 (12)	15 (15)	0.57

*AB* allele burden, *BM* bone marrow, *DIPSS* dynamic international prognostic score system, *HMR* high molecular risk, *IPSS* international prognostic score system, *LCM* left costal margin, *LDH* lactate dehydrogenase, *MIPSS70* mutation-enhanced international prognostic scoring system, *MPN* myeloproliferative neoplasm, *PB* peripheral blood, *PMF* primary myelofibrosis, *Pre-PMF* prefibrotic-PMF, *SD* standard deviation, *WHO* world health organization.

*Notes*: ^║^HMR category is defined as the presence of at least one mutation in any of the following genes: *ASXL1*, *EZH2*, *SRSF2*, or *IDH1/2*. ^†^≥2 HMR mutations indicates the presence of two or more mutated genes among *ASXL1*, *EZH2*, *SRSF2*, and *IDH1/2* (two or more mutations in the same gene are counted as one). Evaluable patients for each variable are reported for prefibrotic/overt PMF, respectively.

## Pre-PMF

In pre-PMF, patients with a CP were more likely to have male gender, older age, higher PB blasts and CD34 + cells, higher serum LDH, higher prevalence of splenomegaly, hepatomegaly, constitutional symptoms, and bone marrow (BM) fibrosis grade 1. Cytogenetic abnormalities and very high risk (VHR) karyotype were more frequent in the CP group. With regards to driver mutations, patients with CP were more likely to be *JAK2*-unmutated and triple negative, with no differences regarding *JAK2* mutant burden. Among non-driver mutations, the cytopenic group was significantly enriched in mutations in *ASXL1*, *N/KRAS*, *U2AF1*, *RUNX1*, *SETBP1*, and *CUX1*, as well as ≥ 2 high molecular risk (HMR; i.e. *ASXL1*, *EZH2*, *IDH1/2*, *SRSF2*) mutations. There were no remarkable differences according to the number of cytopenias (not shown in detail).

After a median follow-up of 76 (95% CI 59–95) months, 76 (35%) deaths were reported, with a median overall survival (OS) of 149 (95% CI 90–205) months. In univariate analysis, pre-PMF patients with CP had a remarkably inferior OS compared to their proliferative counterparts (HR 5.6, 95% CI 3.5–9, *P* <0.0001), with median of 36 (95% CI 26–60) and 193 (95% CI 130–232) months, respectively (Fig. 1B). The number of cytopenias (Supplemental Fig. 1A) and the severity of the CP (Supplemental Fig. 1B) were uninfluential. To dissect the contribution of individual cytopenias with other established prognostic factors, we conducted a multivariate Cox analysis that included leukopenia, severe/moderate anemia and thrombocytopenia, and the variables included in the MIPSS70 score. The final model identified both severe and moderate anemia, leukocytosis, constitutional symptoms and HMR category as independent predictors of inferior OS (Supplemental Table 2).

At the last follow-up, 20 (10%) patients had transformed to acute leukemia. After competing risk analysis, the 5-year cumulative incidence (CuI) of leukemic transformation (LT) was significantly higher in patients with a CP compared to their proliferative counterparts (30%, 95% CI 16–45 and 5%, 95% CI 2–10, respectively; Grey test *P* <0.0001) (Fig. 1C). Neither the number nor the severity of cytopenias affected the rate of LT (Supplemental Fig. 1C, D).

Finally, we aimed at assessing whether the risk of progression to overt PMF was affected by CP. A total of 139 (64%) pre-PMF patients were informative, based on the availability of clinical and/or histologic data defining the progression to overt PMF; of these, 32 (23%) progressed to overtly fibrotic phase. A CP was associated with a significantly shorter fibrotic progression-free survival (PFS; median 33 months, 95% CI 10-not reached) compared the proliferative counterpart (median 193 months, 95% CI 132-not reached) (HR 10.2, 95% CI 4–26.2, *P* <0.0001) (Supplemental Fig. 1E). The 5-year CuI of overt PMF progression, in a competing risk analysis, was significantly higher in pre-PMF patients with a CP compared to their proliferative counterparts (67%, 95% CI 26–89 and 15%, 95% CI 8–24, respectively; Grey test *P* <0.0001) (Fig. 1D). Of note, anemia and thrombocytopenia were significantly more prevalent among pre-PMF patients who progressed to overt-PMF within 5 years from diagnosis (respectively: 26% vs 3%, *P* <0.0001; 16% vs 0%, *P* <0.0001).

## Overt PMF

A CP was associated with older age, higher CD34 + cell count, higher prevalence of BM fibrosis grade 3, lower *JAK2* mutant burden, TN status, and *U2AF1* mutations. Patients with ≥2 cytopenias were more likely to have karyotype abnormalities and mutations in *CBL* and *U2AF1*.

After a median follow-up of 94 (95% CI 79–115) months, 118 (55%) deaths were reported, with a median OS of 65 (95% CI 54–87) months. The OS of patients with CP (median 54 months, 95% CI 44–72) was significantly shorter compared to the proliferative group (median 96 months, 95% CI 64–139) (HR 1.7, 95% CI 1.2–2.4, *P* = 0.0026) (Fig. 1E). Patients harboring ≥ 2 cytopenias had an inferior OS (median 43 months, 95% CI 19–55) compared to patients with one sole cytopenia (median 64 months, 95% CI 45–76) (HR 1.9, 95% CI 1.1–3.2, *P* = 0.0146) (Supplemental Fig. 2A). Remarkably, a severe CP was associated with significantly inferior OS compared to patients with not-severe cytopenias (HR 2.9, 95% CI 1.7–4.8, *P* <0.0001), with median of 28 (95% CI 19–47) and 72 (95% CI 52–91) months, respectively (Supplemental Fig. 2B). Upon multivariate Cox proportional hazards analysis, severe thrombocytopenia, severe anemia, PB blast count ≥ 2%, HMR category and ≥2 HMR mutated genes independently predicted for inferior OS (Supplemental Table 2); severe thrombocytopenia showed the highest HR (5.8, 95% CI 2.5–13.7).

At last follow-up, a total of 28 (14%) patients transformed to acute leukemia. After competing risk analysis, the CuI of LT was not statistically different among cytopenic and proliferative patients, with 5-year rates of 15% (95% CI 8–23) and 12% (95% CI 6–20), respectively (Fig. 1F). The number and severity of cytopenias did not impact the CuI of LT (Supplemental Fig. 2C, D), although there was a trend for patients with severe compared to not-severe cytopenias (5-year CuI of LT 23%, 95% CI 10–38 and 10%, 95% CI 4–20, respectively; Grey test *P* = 0.0719).

In summary, the current study provides a comprehensive analysis of the CP in a large cohort of WHO-defined pre- and overt PMF. We showed that cytopenic features, that are more common in overt than pre-PMF, are associated with distinct high-risk clinical and molecular features predominantly in pre-PMF. Of note, *U2AF1* mutations emerged as a distinct abnormality of CP in both PMF subtypes, suggesting that they might contribute to ineffective hematopoiesis and reinforcing their adverse prognostic role [13, 14]. A CP was associated with inferior OS in both PMF subtypes, and with a higher risk of LT in pre-PMF. While in pre-PMF the adverse prognostic impact of a CP was independent of the number and severity of cytopenias, in overt PMF the impact on OS seemed to be affected mainly by the CP severity, with severe thrombocytopenia having the greatest impact. Finally, we highlighted that a CP is an important risk factor for fibrotic progression in patients with pre-PMF, particularly for those presenting with anemia and thrombocytopenia. Overall, our results further expand the characterization of the cytopenic features in PMF with novel insights as regards the distinction between pre- and overt PMF. Despite the limitations associated with its arbitrary definition, identification of the CP is straightforward, does not require invasive or advanced technologies and, above all, can be performed longitudinally.

Cytopenia represents a significant challenge in the contemporary management of PMF. Currently, there are few agents aimed at treating cytopenic PMF, including immunomodulatory drugs, hypomethylating agents, and JAK inhibitors such as momelotinib and pacritinib, and development of new agents specifically tailored to this patient population remains an unmet need. The association with *U2AF1* mutations may prompt the study of splicing modulators [14].

## Supplementary information

Supplemental Information

## Data Availability

The datasets generated during and/or analysed during the current study are available from the corresponding author on reasonable request.
