# Supplementary material for: Differential prognostic impact of cytopenic phenotype in prefibrotic vs overt primary myelofibrosis
Source: Blood Cancer J. 2022 Aug 12;12(8):116. doi: 10.1038/s41408-022-00713-6 (PMC9374751; doi:10.1038/s41408-022-00713-6)
Supplement: Supplementary file 1 — Supplemental Information [file 41408_2022_713_MOESM1_ESM.pdf]

## Supplemental Information

### Supplemental Contents

Supplemental Methods…………...………………………………..……………………………………….....Pag. 2

Supplemental Tables……………………………………………………………………...…………...….Pag. 3

Supplemental Figures………………………………………………………………………...……..Pag. 6

References……………………………………………………………………………………………..Pag. 10

**Supplemental Methods**

After approval by the Institutional Review Board (IRB) of the University of Florence (Florence, Italy), patients with a diagnosis of either pre- or overt primary myelofibrosis (PMF) were included in the current study. The diagnosis was retrospectively confirmed according to the 2016 revised world health organization (WHO)[1]. Cytopenias at diagnosis were defined as follows: leukopenia for leukocytes $<4\times10^9$/L, sex-adjusted anemia for hemoglobin (Hb) <11 g/dL for male and <10 g/dL for female, and thrombocytopenia for platelets $<100\times10^9$/L. A cytopenic phenotype (CP) was defined by the presence of at least one cytopenia, whereas patients not included in the cytopenic group were considered as having a proliferative phenotype. Sex-adjusted anemia was further categorized as moderate (Hb 9-10.1 and 8-9.9 for male or female, respectively) and severe (Hb <9 g/dL and <8 g/dL for male and female, respectively). Similarly, moderate and severe thrombocytopenia was defined by a platelet count $50\text{-}99\times10^9$/L and $<50\times10^9$/L, respectively. Patients with severe anemia and/or thrombocytopenia were considered as having a severe CP.

Mutational analysis for driver mutations and targeted next generation sequencing (NGS) for 29 myeloid-relevant genes was performed on DNA obtained from peripheral blood (PB) collected at diagnosis or first referral by previously described methods[2]. Statistical analyses considered clinical and laboratory parameters obtained at diagnosis or first referral. Continuous variables were presented as median (range) and categorical variables as frequency (percentage), and compared among categories by the Mann-Whitney test and the $\chi2$ test, respectively. Overall survival (OS) analysis was computed from the date of diagnosis to date of death (uncensored) or last contact (censored). Limited to the pre-PMF cohort, date of progression to overt PMF replaced date of death, as the uncensored variable, for estimating fibrotic progression-free survival (PFS). For this purpose, progression to overt PMF was established by the clinical features consistent with advanced PMF, including worsening of anemia, progressive splenomegaly, overt leukoerythroblastosis and/or histologic documentation BM fibrosis grade 2-3, as previously described[3]. The Kaplan-Meier method was used to prepare OS and PFS curves, which were compared by the log-rank test. Cumulative incidence of leukemic transformation (LT) and progression to overt PMF was calculated after competing risk analysis, and compared between groups using the Gray's test. Cox proportional hazard regression model followed by backward stepwise selection was used for univariate and multivariable analysis of time-to-event data. *P* values less than 0.05 were considered significant. The JMP® Pro 15.2.1 software from SAS Institute (Cary, NC, USA) and R software version 4.1.1 were used for all calculations.

# Supplemental Tables

**Supplemental Table 1.** Clinical and laboratory features of the study population according to disease subtype (pre- *versus* overt PMF)

| | Variable | All patients n=431 | Pre-PMF n=216 (50%) | Overt PMF n=215 (50%) | Pre- *vs* overt PMF *P* value |
|---|---|---|---|---|---|
| Clinical and demographics | Male sex; n (%) | 274 (64) | 118 (55) | 156 (73) | **.0001** |
| | Age at diagnosis, years; median (range) | 64 (18-90) | 62 (18-90) | 65 (21-89) | **.0267** |
| | Leukocytes, x$10^9$/l; median (range) | 8.9 (0.7-250) | 9 (0.7-250) | 8.7 (1.4-90.8) | .27 |
| | Leukocytes <4x$10^9$/l; n (%) | 41 (10) | 12 (6) | 29 (13) | **.0050** |
| | Leukocytes >25x$10^9$/l; n (%) | 46 (11) | 20 (9) | 26 (12) | .34 |
| | Hemoglobin, g/dl; median (range) | 12 (4.2-18.3) | 13.3 (4.7-18.3) | 11 (4.2-16.4) | **<.0001** |
| | Sex-adjusted moderate anemia*; n (%) | 76 (18) | 21 (10) | 55 (26) | **<.0001** |
| | Sex-adjusted severe anemia*; n (%) | 56 (13) | 19 (9) | 37 (17) | **.0094** |
| | Platelets, x$10^9$/l; median (range) | 379 (2-1800) | 518 (2-1800) | 268 (1-1392) | **<.0001** |
| | Platelets <100x$10^9$/l; n (%) | 48 (11) | 18 (8) | 30 (14) | .06 |
| | Platelets <50x$10^9$/l; n (%) | 18 (4) | 7 (3) | 11 (5) | .33 |
| | Platelets >450x$10^9$/l; n (%) | 184 (43) | 124 (57) | 60 (28) | **<.0001** |
| | Peripheral CD34+, %; mean (SD); evaluable=278 | 0.9 (2.2) | 0.4 (0.2) | 1.4 (0.2) | **<.0001** |
| | PB blasts, %; mean (SD); evaluable=423 | 0.7 (2.1) | 0.5 (1.7) | 1 (2.4) | **<.0001** |
| | LDH, U/L; median (range); evaluable=314 | 459 (127-2981) | 335 (127-2643) | 644 (130-2981) | **<.0001** |
| | Splenomegaly (>5 cm below the LCM); n (%); evaluable=419 | 259 (62) | 97 (46) | 162 (78) | **<.0001** |
| | Hepatomegaly; n (%); evaluable=407 | 127 (31) | 49 (24) | 78 (39) | **.0014** |
| | Constitutional symptoms; n (%); evaluable=398 | 120 (30) | 43 (22) | 77 (38) | **.0004** |
| Phenotype | Cytopenic phenotype; n (%) | 155 (36) | 50 (23) | 105 (49) | **<.0001** |
| | Moderate cytopenic phenotype; n (%) | 91 (21) | 28 (13) | 63 (29) | **<.0001** |
| | Severe cytopenic phenotype; n (%) | 64 (15) | 22 (10) | 42 (20) | **<.0001** |
| | Cytopenic phenotype with one sole cytopenia; n (%) | 101 (23) | 35 (16) | 66 (31) | **<.0001** |
| | Cytopenic phenotype with ≥2 cytopenias; n (%) | 54 (13) | 15 (7) | 39 (18) | **<.0001** |
| MPN drivers | *JAK2* mutated; n (%); evaluable=399 | 266 (67) | 137 (70) | 129 (64) | .23 |
| | *JAK2*$^{V617F}$ AB; median (range); evaluable=257 | 39 (1-100) | 37 (1-100) | 43 (5-100) | **.0092** |
| | *JAK2*$^{V617F}$ AB lower quartile; n (%); evaluable=257 | 64 (25) | 24 (19) | 40 (31) | **.0333** |
| | *CALR* mutated; n (%); evaluable=394 | 73 (19) | 33 (17) | 40 (20) | .39 |
| | *MPL* mutated; n (%); evaluable=396 | 22 (6) | 11 (6) | 11 (6) | .96 |
| | Triple negative; n (%); evaluable=393 | 41 (10) | 21 (11) | 20 (10) | .86 |
| | Double mutated; n (%); evaluable=391 | 9 (2) | 6 (3) | 3 (2) | .31 |
| Myeloid neoplasm-associated genes | *ASXL1* mutated; n (%); evaluable=358 | 101 (28) | 27 (15) | 74 (41) | **<.0001** |
| | *CBL* mutated; n (%); evaluable=318 | 18 (6) | 5 (3) | 13 (8) | .06 |
| | *CSF3R* mutated; n (%); evaluable=216 | 2 (1) | 1 (1) | 1 (1) | .97 |
| | *CUX1* mutated; n (%); evaluable=201 | 3 (1) | 1 (1) | 2 (2) | .51 |
| | *DNMT3A* mutated; n (%); evaluable=320 | 20 (6) | 8 (5) | 12 (7) | .42 |
| | *EZH2* mutated; n (%); evaluable=358 | 32 (9) | 4(2) | 28 (15) | **<.0001** |
| | *IDH1/2* mutated; n (%); evaluable=358 | 15 (4) | 1 (1) | 14 (8) | **.0008** |
| | *KIT* mutated; n (%); evaluable=278 | 4 (1) | 3 (2) | 1 (1) | .31 |
| | *NF-E2* mutated; n (%); evaluable=263 | 11 (4) | 5 (4) | 6 (5) | .75 |
| | *N/KRAS* mutated; n (%); evaluable=276 | 25 (9) | 5 (4) | 20 (14) | **.0019** |
| | *RUNX1* mutated; n (%); evaluable=277 | 8 (3) | 2 (1) | 6 (4) | .15 |
| | *SETBP1* mutated; n (%); evaluable=216 | 5 (2) | 3 (3) | 2 (2) | .70 |
| | *SF3B1* mutated; n (%); evaluable=278 | 18 (6) | 6 (4) | 12 (9) | .16 |
| | *SH2B3/LNK* mutated; n (%); evaluable=277 | 11 (4) | 4 (3) | 7 (5) | .39 |
| | *SRSF2* mutated; n (%); evaluable=358 | 38 (11) | 16 (9) | 22 (12) | .36 |
| | *TET2* mutated; n (%); evaluable=320 | 63 (20) | 34 (22) | 29 (18) | .38 |
| | *TP53* mutated; n (%); evaluable=282 | 9 (3) | 4 (3) | 5 (4) | .77 |
| | *U2AF1* mutated; n (%); evaluable=278 | 14 (5) | 1 (1) | 13 (9) | **.0012** |

| | | | | | |
|---|---|---|---|---|---|
| | *ZRSR2* mutated; n (%); evaluable=216 | 17 (8) | 10 (9) | 7 (7) | .52 |
| | HMR mutations[‖]; n (%); evaluable=358 | 128 (36) | 35 (20) | 93 (51) | **<.0001** |
| | ≥2 HMR mutations[†]; n (%); evaluable=358 | 51 (14) | 12 (7) | 39 (21) | **<.0001** |
| **Cytogenetics** | Abnormal karyotype; n (%); evaluable=299 | 87 (29) | 38 (23) | 49 (36) | **.0159** |
| | Favorable karyotype; n (%) | 247 /83) | 142 (87) | 105 (77) | |
| | Unfavorable karyotype; n (%) | 34 (11) | 12 (7) | 22 (17) | **.0475** |
| | Very high risk karyotype; n (%) | 18 (6) | 9 (6) | 9 (7) | |
| **Prognostic stratification** | IPSS risk stratification; evaluable=388 | | | | |
| | Low risk; n (%) | 131 (34) | 88 (46) | 43 (22) | |
| | Intermediate-1 risk; n (%) | 113 (29) | 61 (32) | 52 (27) | **<.0001** |
| | Intermediate-2 risk; n (%) | 68 (18) | 19 (10) | 49 (25) | |
| | High risk; n (%) | 76 (20) | 25 (13) | 51 (26) | |
| | DIPSS risk stratification; evaluable=388 | | | | |
| | Low risk; n (%) | 131 (34) | 88 (46) | 43 (22) | |
| | Intermediate-1 risk; n (%) | 150 (39) | 74 (38) | 76 (39) | **<.0001** |
| | Intermediate-2 risk; n (%) | 88 (23) | 27 (14) | 61 (31) | |
| | High risk; n (%) | 19 (5) | 4 (2) | 15 (8) | |
| | MIPSS70 risk stratification; evaluable=343 | | | | |
| | Low risk; n (%) | 109 (32) | 99 (58) | 10 (6) | |
| | Intermediate risk; n (%) | 145 (42) | 53 (31) | 92 (54) | **<.0001** |
| | High risk; n (%) | 89 (26) | 20 (12) | 69 (40) | |
| | Deaths; n (%) | 194 (45) | 76 (35) | 118 (55) | **<.0001** |
| | Leukemic transformation; n (%) | 48 (12) | 20 (10) | 28 (14) | .23 |

*Notes*: [*]Sex-adjusted moderate anemia was defined as a hemoglobin level between 9-10.9 g/dL for male and 8-9.9 g/dL for female; sex-adjusted severe anemia was defined as a hemoglobin level <9 g/dL for male and <8 g/dL for female. [‖]HMR category is defined as the presence of at least 1 mutation in any of the following genes: *ASXL1*, *EZH2*, *SRSF2*, or *IDH1/2*. [†]≥2 $HMR^{MT}$ indicates the presence of two or more mutated genes among *ASXL1*, *EZH2*, *SRSF2*, and *IDH1/2* (two or more mutations in the same gene are counted as one).

*Abbreviations:* AB: allele burden; BM: bone marrow; DIPSS: dynamic international prognostic score system; HMR: high molecular risk; IPSS: international prognostic score system; LCM: left cost margin; LDH: lactate dehydrogenase; MIPSS: mutation-enhanced international prognostic scoring system; MPN: myeloproliferative neoplasm; PB: peripheral blood; PMF: primary myelofibrosis; Pre-PMF: prefibrotic-PMF; SD: standard deviation.

**Supplemental Table 2.** Results of univariate and multivariate Cox proportional hazards model of OS in prefibrotic and overt PMF

| **Covariate** | **Prefibrotic PMF** | | | | **Overt PMF** | | | |
|---|---|---|---|---|---|---|---|---|
| | **Univariate** | | **Multivariate** | | **Univariate** | | **Multivariate** | |
| | **HR (95% CI)** | ***P* value** | **HR (95% CI)** | ***P* value** | **HR (95% CI)** | ***P* value** | **HR (95% CI)** | ***P* value** |
| Leukopenia<br>WBC <4 × $10^9$/L | 2.3 (1.1-4-7) | **.0333** | | | 1.0 (0.7-1.6) | 0.9 | NS | |
| Leukocytosis<br>WBC >25 × $10^9$/L | 7.9 (4.6-13-3) | **<.0001** | 7.1 (3.3-15.2) | **<.0001** | 3.1 (1.8-5.2) | **<.0001** | NS | |
| Moderate anemia<br>Hb 9-10-9 (♂)/8-9.9 g/dL (♀) | 3.5 (1.9-6.4) | **<.0001** | 3.8 (1.7-8.2) | **.0008** | 1.1. (0.7-1.7) | 0.6 | NS | |
| Severe anemia<br>Hb <9 (♂)/<8 g/dL (♀) | 3.4 (1.8-6.2) | **<.0001** | 4 (1.8-8.6) | **.0004** | 2.6 (1.7-4.1) | **<.0001** | 2.4 (1.4-4.1) | **.0020** |
| Moderate thrombocytopenia<br>Plt 50-99 × $10^9$/L | 2.7 (1.3-5.7) | **.0075** | NS | | 2.1 (1.1-3.8) | **.0250** | NS | |
| Severe thrombocytopenia<br>Plt <50 × $10^9$/L | 4.1 (1.8-9.5) | **.0010** | NS | | 5.4 (2.8-10.6) | **<.0001** | 5.8 (2.5-13.7) | **<.0001** |
| PB blasts ≥2% | 9.3 (5.3-16.1) | **<.0001** | NS | | 3 (2-4.7) | **<.0001** | 2.1 (1.3-3.5) | **.0037** |
| BM fibrosis grade ≥2 | Not applicable | | Not applicable | | Not applicable | | Not applicable | |
| Constitutional symptoms | 3.2 (1.9-5.3) | **<.0001** | 2.5 (1.4-4.3) | **.0014** | 1.7 (1.1-2.5) | **.0102** | NS | |
| Absence of *CALR* type 1/like | 7.5-54.8 | **.0046** | NS | | 3.3 (1.6-6.9) | **.0012** | NS | |
| HMR[‖] category | 4.9 (2.8-8.6) | **<.0001** | 3.8 (2.1-6.8) | **<.0001** | 2.7 (1.8-4.3) | **<.0001** | 2.1 (1.3-3.5) | **.0037** |
| ≥2 HMR[†] mutated genes | 11 (5.5-22) | **<.0001** | NS | | 2.8 (1.8-4.5) | **<.0001** | 2.2 (1.3-3.7) | **.0036** |

*Notes*: [‖]HMR category is defined as the presence of at least 1 mutation in any of the following genes: *ASXL1*, *EZH2*, *SRSF2*, or *IDH1/2*. [†]≥2 HMR$^{MT}$ indicates the presence of two or more mutated genes among *ASXL1*, *EZH2*, *SRSF2*, and *IDH1/2* (two or more mutations in the same gene are counted as one).
*Abbreviations*: BM: bone marrow; CI: confidence interval; Hb: hemoglobin; HR: hazard ratio; HMR: high molecular risk; NA: not applicable; NS: not significant; OS: overall survival; PB: peripheral blood; Plt: platelets; PMF: primary myelofibrosis; WBC: white blood cell.

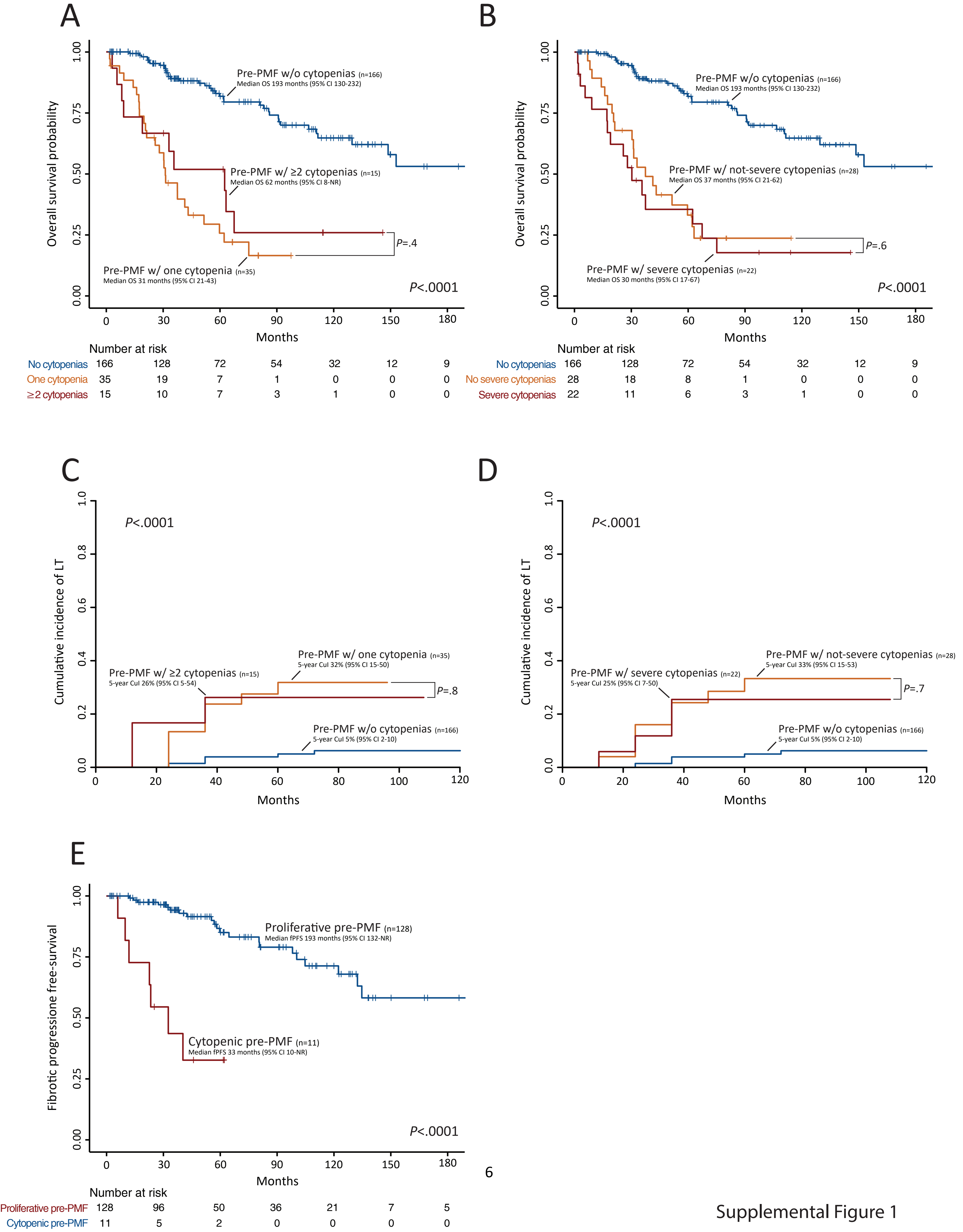


Supplemental Figure 1

**Supplemental Figure 1. A.** Kaplan-Meier estimates of overall survival in patients with pre-PMF according to the presence and number of cytopenias (no *vs* one *vs* ≥2 cytopenias). **B.** Kaplan-Meier estimates of overall survival in patients with pre-PMF according to the presence and severity of cytopenias. **C.** Competing risks-adjusted estimates of cumulative incidence of leukemic transformation in pre-PMF according to the presence and number of cytopenias (no *vs* one *vs* ≥2 cytopenias). **D.** Competing risks-adjusted estimates of cumulative incidence of leukemic transformation in pre-PMF according to the presence and severity of cytopenias. **E.** Kaplan-Meier estimates of fibrotic progression-free survival in 139 pre-PMF patients according to disease phenotype (cytopenic *vs* proliferative). *Abbreviations*: CI: confidence interval; CuI: cumulative incidence; fPMF: fibrotic progression-free survival; LT: leukemic transformation; NR: not reached; OS: overall survival; pre-PMF: prefibrotic primary myelofibrosis; w/: with; w/o: without.

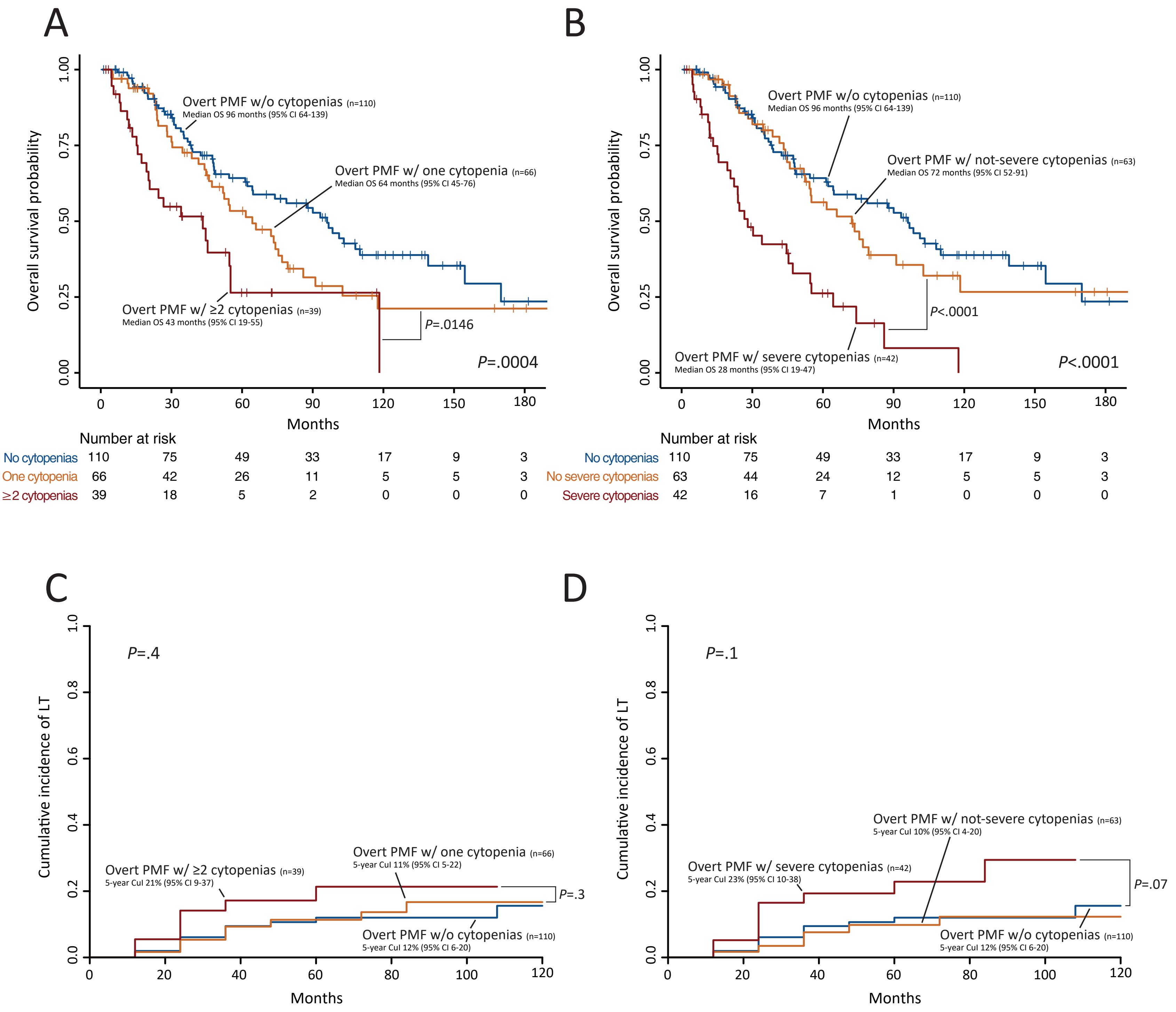




Supplemental Figure 2

**Supplemental Figure 2. A.** Kaplan-Meier estimates of overall survival in patients with overt PMF according to the presence and number of cytopenias (no *vs* one *vs* ≥2 cytopenias). **B.** Kaplan-Meier estimates of overall survival in patients with overt PMF according to the presence and severity of cytopenias. **C.** Competing risks-adjusted estimates of cumulative incidence of leukemic transformation in overt PMF according to the presence and number of cytopenias (no *vs* one *vs* ≥2 cytopenias). **D.** Competing risks-adjusted estimates of cumulative incidence of leukemic transformation in overt PMF according to the presence and severity of cytopenias. *Abbreviations*: CI: confidence interval; CuI: cumulative incidence; LT: leukemic transformation; OS: overall survival; PMF: primary myelofibrosis; w/: with; w/o: without.

**References**


1. Arber DA, Orazi A, Hasserjian R, et al. The 2016 revision to the World Health Organization (WHO) classification of myeloid neoplasms and acute leukemia. *Blood.* 2016:blood-2016-2003-643544.
2. Guglielmelli P, Lasho TL, Rotunno G, et al. MIPSS70: mutation-enhanced international prognostic score system for transplantation-age patients with primary myelofibrosis. *Journal of Clinical Oncology.* 2017;36(4):310-318.
3. Carobbio A, Guglielmelli P, Rumi E, et al. A multistate model of survival prediction and event monitoring in prefibrotic myelofibrosis. *Blood cancer journal.* 2020;10(10):1-7.
